# Effect of statin use on head and neck cancer prognosis in a multicenter study using a Common Data Model

**DOI:** 10.1038/s41598-023-45654-7

**Published:** 2023-11-13

**Authors:** Soobeen Seol, Jung Ran Choi, Byungjin Choi, Sungryeal Kim, Ja Young Jeon, Ki Nam Park, Jae Hong Park, Min Woo Park, Young-Gyu Eun, Jung Je Park, Byung-Joo Lee, Yoo Seob Shin, Chul-Ho Kim, Rae Woong Park, Jeon Yeob Jang

**Affiliations:** 1 Department of Biomedical Sciences, Ajou University Graduate School of Medicine, 164 World cup-ro Yeongtong-gu, Suwon-si, Gyeonggi-do 16499 Republic of Korea; 2 Department of Otolaryngology, Ajou University School of Medicine, Ajou University Hospital, 164 World cup-ro Yeongtong-gu, Suwon-si, Gyeonggi-do 16499 Republic of Korea; 3 Department of Otorhinolaryngology-Head and Neck Surgery, Inha University College of Medicine, Incheon, Republic of Korea; 4 Department of Endocrinology and Metabolism, Ajou University School of Medicine, Suwon, Republic of Korea; 5 Department of Otorhinolaryngology-Head and Neck Surgery, Soonchunhyang University, Bucheon, Republic of Korea; 6 Department of Otorhinolaryngology-Head and Neck Surgery, Soonchunhyang University Cheonan Hospital, Cheonan, Republic of Korea; 7 Department of Otolaryngology-Head and Neck Surgery, Kangdong Sacred Heart Hospital, Seoul, Republic of Korea; 8 Department of Otolaryngology-Head and Neck Surgery, School of Medicine, Kyung Hee University, Kyung Hee University Medical Center, Seoul, Republic of Korea; 9 Department of Otorhinolaryngology, College of Medicine, Gyeongsang National University and Hospital, Jinju, Republic of Korea; 10 Institute of Health Sciences, Gyeongsang National University, Jinju, Republic of Korea; 11 Department of Otorhinolaryngology-Head and Neck Surgery, College of Medicine, Pusan National University and Biomedical Research Institute, Pusan National University Hospital, Busan, Republic of Korea; 12 Department of Biomedical Informatics, Ajou University School of Medicine, 164 World cup-ro Yeongtong-gu, Suwon-si, Gyeonggi-do 16499 Republic of Korea

**Keywords:** Diseases, Medical research

## Abstract

Few studies have found an association between statin use and head and neck cancer (HNC) outcomes. We examined the effect of statin use on HNC recurrence using the converted Observational Medical Outcome Partnership (OMOP) Common Data Model (CDM) in seven hospitals between 1986 and 2022. Among the 9,473,551 eligible patients, we identified 4669 patients with HNC, of whom 398 were included in the target cohort, and 4271 were included in the control cohort after propensity score matching. A Cox proportional regression model was used. Of the 4669 patients included, 398 (8.52%) previously received statin prescriptions. Statin use was associated with a reduced rate of 3- and 5-year HNC recurrence compared to propensity score-matched controls (risk ratio [RR], 0.79; 95% confidence interval [CI], 0.61–1.03; and RR 0.89; 95% CI 0.70–1.12, respectively). Nevertheless, the association between statin use and HNC recurrence was not statistically significant. A meta-analysis of recurrence based on subgroups, including age subgroups, showed similar trends. The results of this propensity-matched cohort study may not provide a statistically significant association between statin use and a lower risk of HNC recurrence. Further retrospective studies using nationwide claims data and prospective studies are warranted.

## Introduction

Head and neck cancer (HNC) is the sixth most common type of malignancy, with a high morbidity and 5-year survival rates ranging from 31.9 to 89.5% depending on different cancer sites^1–3^. More than 90% of cases of HNC are classified as squamous cell carcinomas, which usually occur from the mucosal lining of the aerodigestive tract starting in the nasal cavity and ending in the throat at the larynx, with other sites such as the oral cavity and hypopharynx^2,4,5^. Although efficient prevention procedures and treatment for this malignancy have been enhanced, there are no evidence-based prevention strategies for HNC, except for smoking cessation^2,6^. Novel strategies for the prevention of HNC to decrease disease burden involve the discovery of novel risk factors and repurposing existing drugs^7,8^.

Statins are commonly prescribed medications primarily used to lower cholesterol^9^ and show anti-cancer effects, particularly anti-inflammatory and immunomodulatory effects^10,11^. Cholesterol may be an important factor in cancer development or progression because it is involved in diverse pathways involved in carcinogenesis^12^. Regardless of the low cholesterol levels, statins have also been investigated to inhibit cancer cell invasion, reduce proliferation, and elevate apoptosis in neoplastic cells^13–15^. The inflammatory and immunomodulatory effects of cancer often depend on the type of cancer, whether there is a presence of inflammatory markers, and what combination of these markers is beneficial or harmful to cancer prognosis^2,16^. Few studies have suggested that certain inflammatory markers, including tumor-infiltrating lymphocytes, may improve HNC outcomes, whereas some, such as pro-inflammatory cytokine IL-6, may worsen HNC outcomes^2,17–19^. Although there have been no studies on the production of inflammatory markers and statin use in patients with HNC, some studies have examined statin use and the presence of inflammatory biomarkers in the general population and patients with hypercholesterolemia. Various studies have reported a decrease in circulating pro-inflammatory cytokines in statin users^20–22^. Several studies have examined the association between statin use and cancer outcomes in separate tumor sites and have demonstrated protective associations; however, some studies are limited in that they were unable to determine whether there is an association between statin use and HNC outcomes^2,23–28^. However, few studies have demonstrated not only a protective effect between statin exposure and HNC progress but also a protective association between statin intake and overall death and disease-specific death^29–32^.

The Observational Medical Outcomes Partnership (OMOP) Common Data Model (CDM) translates various healthcare data into a standardized format to allow for large-scale analysis^33^. The Observational Health Data Science and Informatics (OHDSI) program is an international attempt to optimize analytic services for a large network of health databases^34^. OMOP-CDM guarantees the homogeneous storage of observational healthcare data across different databases, with interoperable formats and standard terminologies^35^. The terminologies for diagnoses/conditions, observations, and drugs within the OMOP-CDM are founded on, for example, the International Classification of Diseases (ICD) codes, Systematized Nomenclature of Medicine-Clinical Terms (SNOMED-CT)^36^, and normalized naming system for generic and branded drugs (RxNorm). We herein used the OHDSI tool OMOP-CDM to conduct a large retrospective study and identified the association between statin use and recurrence in patients with HNC using propensity score matching. Our primary objective was to examine the impact of statin medication on the overall recurrence of head and neck cancer using the OMOP-CDM real-world database. This study aims to provide deeper insights into the association between statins and head and neck cancer by investigating the influence of statin medication on the clinical outcome (3-or 5-year recurrence) within a substantial population-based study. In this pursuit, the utilization of the OMOP-CDM real-world database was deemed essential to access relevant data.

## Results

### Study population

**Figure 1:**
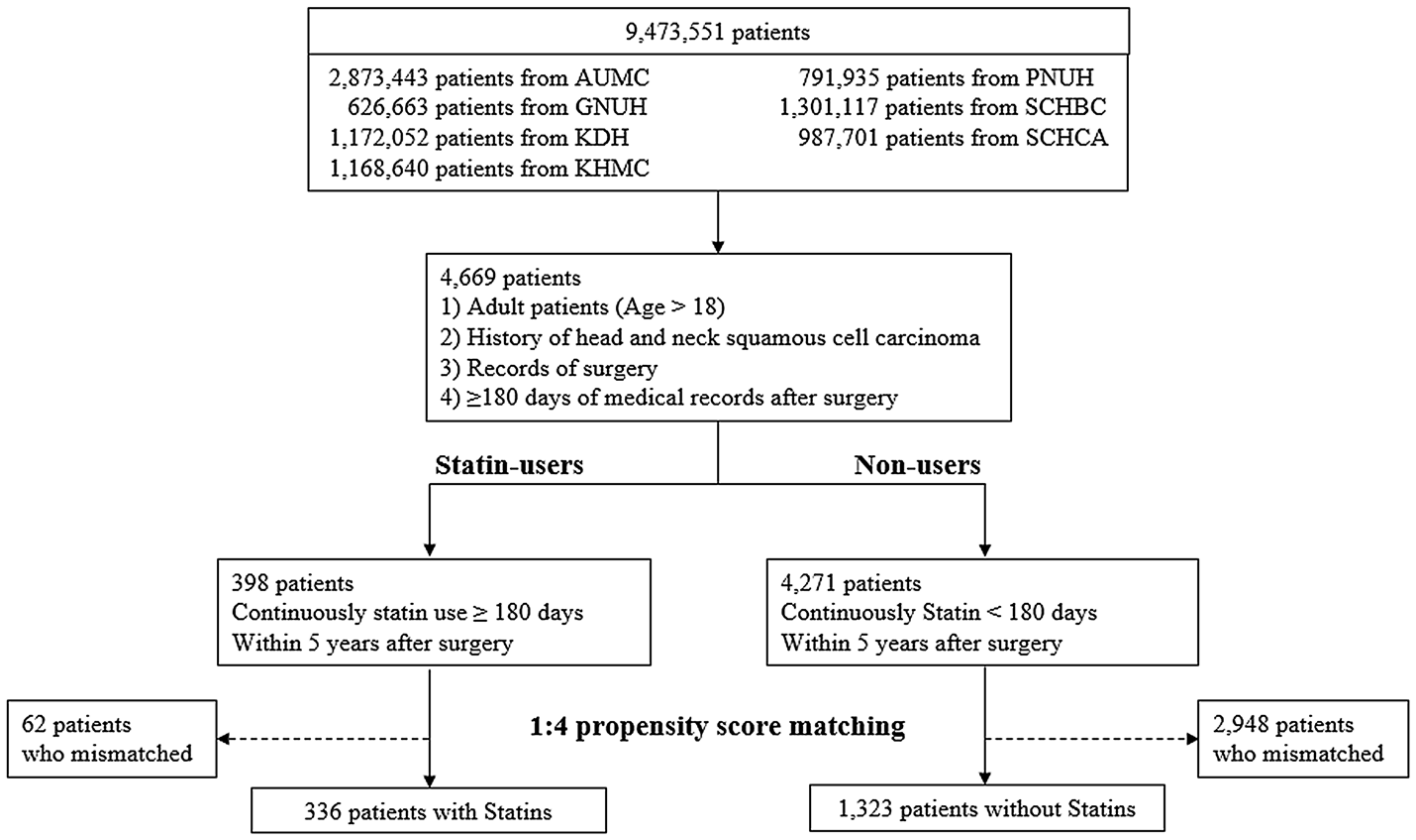
Attrition diagram of the study populations. Schematic diagram of cohort construction. A total of 4,669 participants and 336 statin users were matched with 1,323 statin non-user participants using 1:4 propensity score matching. *AUMC* Ajou University Medical Center, *GNUH* Gyeongsang National University Hospital, *KDH* Kangdong Sacred Heart Hospital, *KHMC* Kyunghee University Medical Center, *PNUH* Pusan National University Hospital, *SCHBC* Soonchunhyang University Bucheon Hospital, *SCHCA* Soonchunhyang University Cheonan Hospital.

We included 4669 patients with HNC from 7 tertiary hospitals in the Republic of Korea. Of these patients, 398 took statin (8.52%). After 1:4 propensity score matching, we selected 336 statin users (target cohort) and 1,323 non-users (control cohort) (Fig. 1). In every hospital only except AUMC, *p*-values from scaled Schoenfeld residuals were higher than 0.05 which verified proportional hazard assumption . As a result, most hospitals performed testing on the proportional hazards residuals. We added results as Supplementary Table 2. Table 1 shows the aggregated baseline characteristics of patients before and after propensity score adjustment across seven hospitals. Regardless of whether the study was conducted before or after propensity score matching, there were significantly more men than women with HNC. For both statin users and non-users, the highest proportion were aged 60–69 years. Statin users had a higher incidence of hypertension, acute myocardial infarction, and cerebrovascular disease, regardless of the propensity score matching.

**Table 1:** Baseline characteristics of target, comparator cohorts.

	Before PS adjustment			After PS adjustment		
	Stain-users (n = 398)	Non-users (n = 4268)	SMD	Stain-users (n = 336)	Non-users (n = 1323)	SMD
Age group						
18–19	0 (0.0%)	6 (0.1%)	− 0.053	0 (0.0%)	2 (0.2%)	− 0.055
20–29	0 (0.0%)	55 (1.3%)	− 0.162	0 (0.0%)	17 (1.3%)	− 0.161
30–39	6 (1.5%)	143 (3.4%)	− 0.120	6 (1.7%)	33 (2.5%)	− 0.049
40–49	20 (5.0%)	508 (11.9%)	− 0.249	17 (5.1%)	140 (10.6%)	− 0.207
50–59	114 (28.6%)	1148 (26.9%)	0.039	103 (30.7%)	359 (27.1%)	0.078
60–69	121 (30.4%)	1321 (31.0%)	− 0.011	104 (31.0%)	419 (31.7%)	− 0.015
70–79	109 (27.4%)	855 (20.0%)	0.176	86 (25.6%)	281 (21.2%)	0.103
80–89	27 (6.8%)	217 (5.1%)	0.072	19 (5.6%)	65 (4.9%)	0.033
90–99	1 (0.3%)	18 (0.4%)	− 0.029	1 (0.3%)	7 (0.5%)	− 0.036
Gender						
Female	56 (14.1%)	755 (17.7%)	− 0.099	50 (14.9%)	212 (16.0%)	− 0.032
Male	342 (85.9%)	3516 (82.3%)	0.099	286 (85.1%)	1111 (84.0%)	0.032
Charlson comorbidity index						
Hypertension	173 (43.5%)	744 (17.4%)	0.590	137 (40.8%)	264 (20.0%)	0.464
Acute myocardial infarction	23 (5.8%)	10 (0.2%)	0.329	16 (4.8%)	2 (0.2%)	0.301
Ongestive heart failure	13 (3.3%)	25 (0.6%)	0.196	10 (3.0%)	11 (0.8%)	0.157
Peripheral vascular disease	4 (1.0%)	14 (0.3%)	0.083	2 (0.6%)	3 (0.2%)	0.058
Cerebrovascular disease	25 (6.3%)	45 (1.1%)	0.281	19 (5.7%)	16 (1.2%)	0.246
Dementia	9 (2.3%)	18 (0.4%)	0.160	4 (1.2%)	7 (0.5%)	0.072
Chronic pulmonary disease	47 (11.8%)	518 (12.1%)	− 0.010	39 (11.6%)	168 (12.7%)	− 0.033
Rheumatologic disease	1 (0.3%)	8 (0.2%)	0.014	1 (0.3%)	5 (0.4%)	− 0.014
Peptic ulcer disease	38 (9.5%)	379 (8.9%)	0.023	34 (10.1%)	120 (9.1%)	0.036
Mild liver disease	4 (1.0%)	84 (2.0%)	− 0.080	3 (0.9%)	36 (2.7%)	− 0.137
Diabetes	85 (21.4%)	326 (7.6%)	0.397	69 (20.5%)	101 (7.6%)	0.377
Diabetes with chronic complications	56 (14.1%)	109 (2.6%)	0.426	31 (9.2%)	37 (2.8%)	0.273
Hemoplegia or paralegia	0 (0.0%)	5 (0.1%)	− 0.048	0 (0.0%)	1 (0.1%)	− 0.039
Renal disease	32 (8.0%)	99 (2.3%)	0.260	20 (6.0%)	38 (2.9%)	0.150
Any malignancy	388 (97.5%)	4217 (98.7%)	− 0.092	328 (97.6%)	1308 (98.9%)	− 0.095
Moderate to severe liver disease	0 (0.0%)	30 (0.7%)	− 0.119	0 (0.0%)	14 (1.1%)	− 0.146
Metastatic solid tumor	90 (22.6%)	1077 (25.2%)	− 0.061	81 (24.1%)	321 (24.3%)	− 0.004
AIDS	1 (0.3%)	2 (0.0%)	0.053	1 (0.3%)	1 (0.1%)	0.051

We used the Standardized Mean Difference (SMD) to compare before and after propensity score matching. SMD, a recently adopted epidemiological metric, quantifies standardized mean discrepancy. Increasing SMD suggests greater group disparity. After matching, the mean SMD reduced by about 0.030, indicating improved covariate matching. An SMD < 0.2 implies an insignificant inter-group difference.

*PS* Propensity score, *SMD* Standardized mean difference.

### Main outcomes

**Figure 2:**
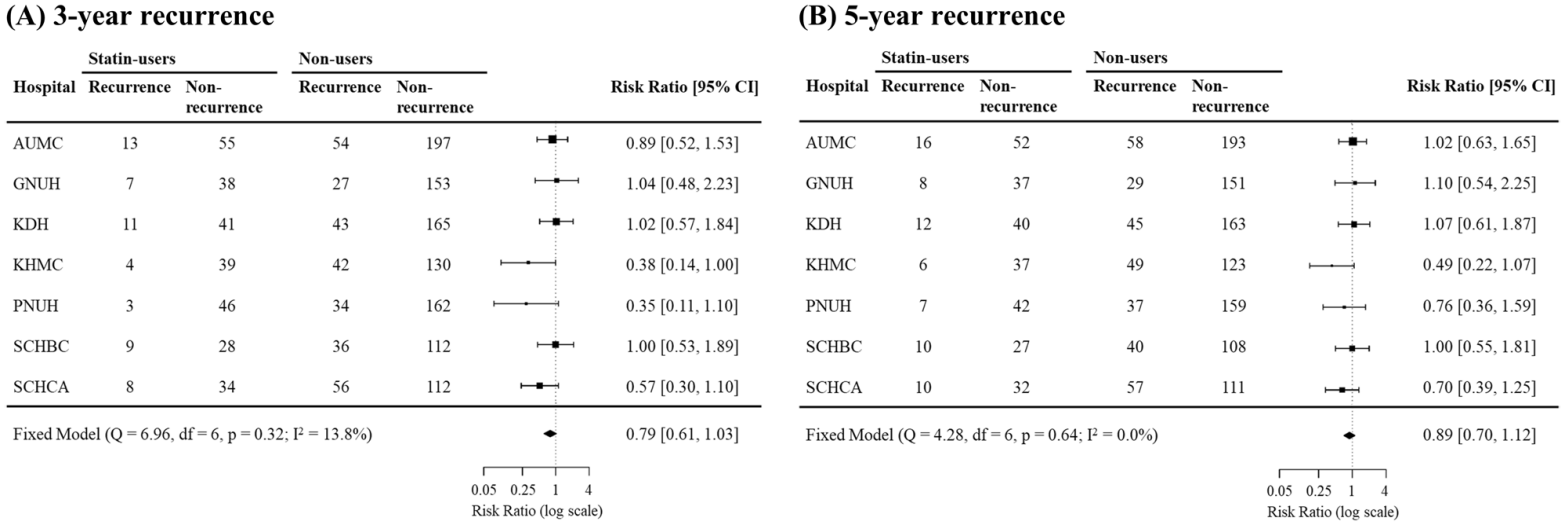
Meta-analysis of statin use and HNC recurrence among statin users and non-users. Forest plots showing multivariable Cox proportional hazards models of statin use and HNC outcomes. Hazard ratios (95% confidence intervals) of (**A**) 3-year recurrence and (**B**) 5-year recurrence for statin use among patients with HNC and controls. *AUMC* Ajou University Medical Center, *GNUH* Gyeongsang National University Hospital, *KDH* Kangdong Sacred Heart Hospital, *KHMC* Kyunghee University Medical Center, *PNUH* Pusan National University Hospital, *SCHBC* Soonchunhyang University Bucheon Hospital, *SCHCA* Soonchunhyang University Cheonan Hospital.

**Figure 3:**
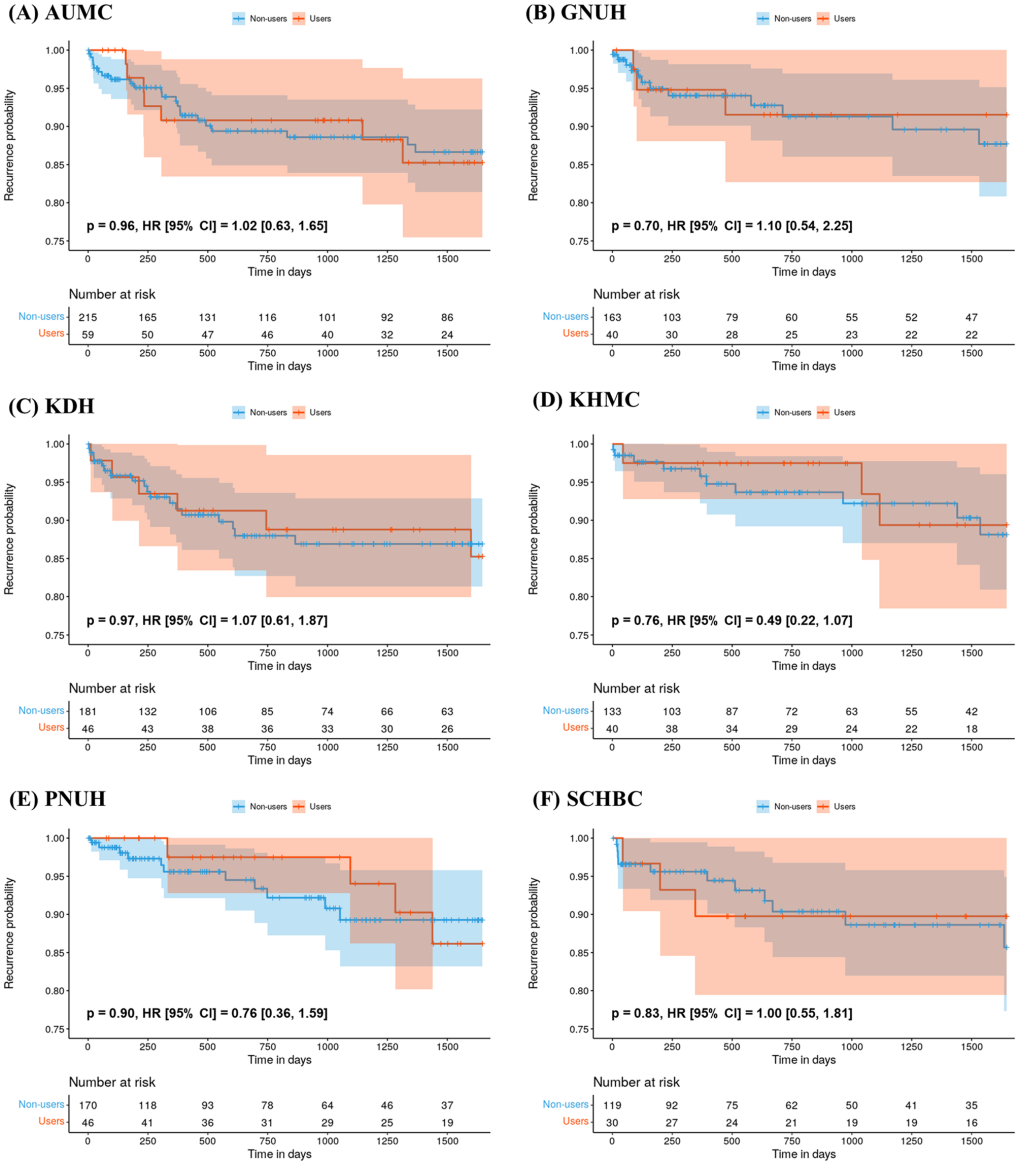

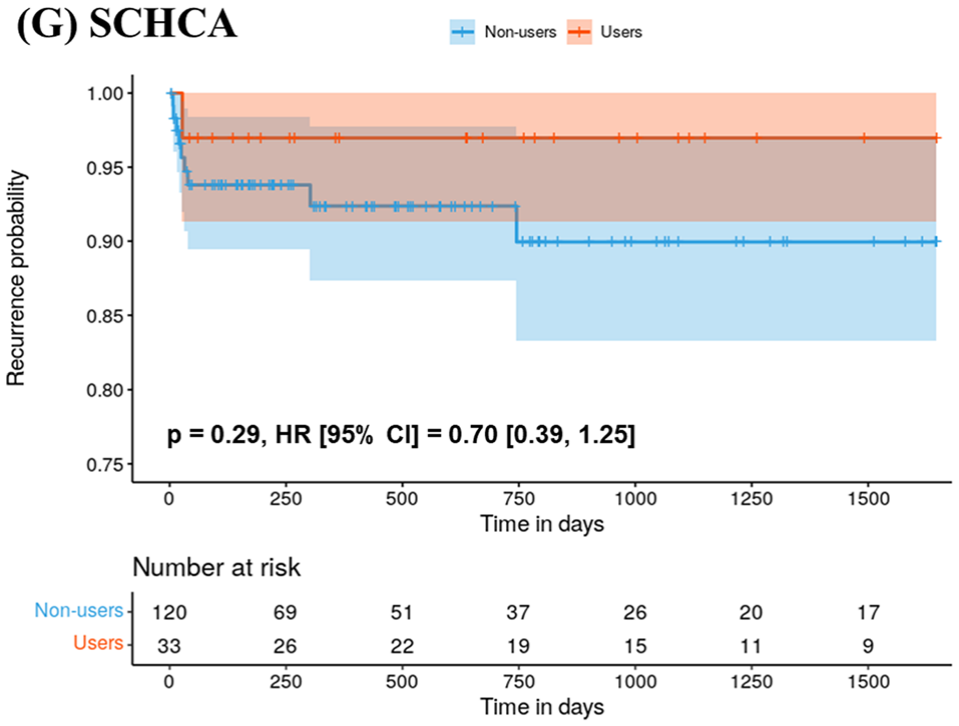
Kaplan–Meier plot showing the risk of 5-year recurrence among statin users and non-users as a function of time. Each alphabet means Kaplan–Meier plot in (**A**) Ajou University Medical Center, AUMC, (**B**) Gyeongsang National University Hospital, GNUH, (**C**) Kangdong Sacred Heart Hospital, KDH, (**D**) Kyunghee University Medical Center, KHMC, (**E**) Pusan National University Hospital, PNUH (**F**) Soonchunhyang University Bucheon Hospital, SCHBC and (**G**) Soonchunhyang University Cheonan Hospital, SCHCA. In the Kaplan–Meier plot, the y-axis represents the recurrence probability, and the x-axis represents time in days. The shaded area represents a 95% confidence interval. *P* value less than 0.05 indicated statistically significant.

Figure 2 shows the forest plot of the risk of 3-year recurrence (a) and 5-year recurrence (b) between statin users and non-users with HNC across the seven databases. We report a meta-analysis based on a fixed-effects model. There was no significant heterogeneity in each database (3-year: Q, 6.96; *p* = 0.32; and *I*^2^, 1.38%; and 5-year: Q, 4.28; *p* = 0.64; and *I*^2^ = 0.0%). The meta-analysis indicated that there was no statistically significant difference in the risk of recurrence at 3 (risk ratio [RR], 0.79; and 95% confidence interval [CI] 0.61–1.03) and 5 years (RR 0.89; 95% CI 0.70–1.12) between statin users and non-users, although there was a tendency for a lower risk ratio in the statin user group. Kaplan–Meier curves were constructed for the risk of recurrence among statin users and non-users in each database (Fig. 3). There was no significant difference in the recurrence rate between both groups, as indicated by the high *p*-value (*p* > 0.05).

The RR for the 3-year and 5-year overall death were lower at 0.71 (95% CI 0.45–1.12) and 0.85 (95% CI 0.57–1.26), respectively, in the statin user group than in the non-user group. However, the association between statin use and mortality was not statistically significant (Supplementary Fig. 1).

### Subgroup outcomes

Table 2 shows the results of the meta-analysis, which found no statistically significant difference in the risk of recurrence between the statin user and non-user subgroups. The exclusion of cases of esophageal cancer did not significantly affect recurrence risk (3-year: RR 0.81. 95% CI 0.53–1.23; and 5-year: RR 0.90; 95% CI 0.68–1.18). Additionally, the association between statin use and risk of recurrence remained non-significant across all age groups (3-year: RR 1.06; 95% CI 0.53–2.11; and 5-year: RR 0.79; 95% CI 0.58–1.09). In the statin subgroups, there was no significant association in the 5-year recurrence rate of HNC (atorvastatin: RR 1.04; 95% CI 0.75–1.43; and rosuvastatin: RR 0.99; 95% CI 0.66–1.48).

**Table 2:** Meta-analysis of recurrence between statin users and non-users, as stratified by subgroups.

	Risk ratio	95% Confidence interval
3-year recurrence		
Excluding esophageal cancer	0.81	0.53–1.23
Elder (> 65)^*R*^	1.06	0.53–2.11
Male	0.87	0.66–1.15
Statin		
Atorvastatin	0.94	0.64–1.37
Rosuvastatin	0.95	0.59–1.51
5-year recurrence		
Excluding esophageal cancer	0.90	0.68–1.18
Elder (> 65)	0.79	0.58–1.09
Male	0.88	0.69–1.14
Statin		
Atorvastatin	1.04	0.75–1.43
Rosuvastatin	0.99	0.66–1.48

*R* Random model.

## Discussion

In this multicenter, observational, retrospective, comparative cohort study of patients with HNC, we demonstrated that there was no significant inverse association between statin use and HNC recurrence compared to propensity score-matched controls. To the best of our knowledge, this study is the first to investigate the association between statin use and HNC recurrence using the OMOP-CDM in Korea.

Several previous studies have suggested an association between statin use and cancer morbidity and mortality^1,2,37,38^. Statins interrupt the rate-limiting enzyme 3-hydroxy-3-methyl-glutaryl-coenzyme A reductase in the cholesterol synthesis pathway^39^, which influences protein synthesis, cell signaling, and cell cycle progression^40^. Statins have also been associated with the elevated production of T-cells in mice and lung tumor cell lines, and the presence of T-cells in tumors is often associated with better outcomes among patients with HNC compared to outcomes in non-users^41^. Therefore, anti-inflammatory and immune modulation are reasonable mechanisms by which statins may offer protection against adverse outcomes in patients with HNC.

Recently, Lebo et al. demonstrated improved overall survival (OS) and disease-specific survival in patients with squamous cell carcinoma of the larynx, hypopharynx, and nasopharynx, who incidentally took statins at the time of diagnosis^32^. Gupta A et al. suggested an association between statin use in patients with HNC with hyperlipidemia and overall mortality in patients with HNC with multiple sites (oral cavity, oropharynx, and other)^29^. Most of the results were similar to those of Lebo et al.^32^, who showed that statin use might reduce the risk of pharynx cancer-related, cervical cancer-related, and larynx cancer-related mortality in a nationwide population^30^. The relevant findings using a matched population also observed an inverse association between statin use and pharynx cancer-related death^30^. Similar to the previous studies, we realized an inverse association between statin use and recurrence in the matched population using OMOP-CDM; however, the association between statin use and HNC recurrence was not statistically significant.

A specific strength of this study was the use of a nationwide population-based database in Korea. The large sample size of the OMOP-CDM provides a large enough sample with sufficient statistical power to identify the association between statin use and HNC recurrence. In addition, using this population-based database with a propensity score matching design can eliminate the selection bias of the findings. Second, the database used in this study was a delegate of the entire Korean population. Most participants recruited in this study were of Korean ethnicity. Thus, the homogeneity of the study sample may have warranted our research because of confounding by ethnicity. Third, we utilized diagnoses and medical records in the OMOP CDM depending on patient recall. This could have prevented recall bias, which frequently occurs in case–control studies.

Nevertheless, this study has several limitations. First, some factors related to HNC were not available in the OMOP CDM, including diet, body mass index, family history, accurate smoking history, and drinking status. These are all potential risk factors for HNC and may influence the association between statin use and HNC recurrence. Second, the database used in this study did not contain records regarding cancer staging (e.g., T/N/M classification), histological examinations, or pathological data. Therefore, we could not evaluate the potential effects of these factors. Third, the data used in this study did not include data on statin compliance. Fourth, the possibility of surveillance bias must be reflected in this observational study. In general, statin users visit physicians and receive medical services more often. It is probable that statin users with underlying HNC had a better likelihood of being diagnosed, whereas those in the control group might have remained symptomless. Thus, surveillance bias did not affect the results of this study. Although we used a propensity matching strategy to reduce bias, it is still probable that the bias can continue in this study.

In conclusion, this population-based study observed an inverse association between statin use and HNC recurrence. However, statin use was not associated with a reduced risk of HNC cancer recurrence. Further epidemiological studies are required to confirm the association between statin use and HNCs in different ethnic groups. Furthermore, Asian and Western populations are recognized to differ in genetic and environmental factors^4,31^. These factors are considered risk factors for HNC and may affect the actual association between statin use and HNC^31^.

## Methods

### Data sources

This multicenter, observational, retrospective, comparative cohort study included seven tertiary hospitals in the Republic of Korea. The study included real-world clinical data of 9,473,551 patients from seven electronic health data (EHR) databases in Korea. All databases were standardized, de-identified into the standard vocabulary of the OMOP-CDM, and stored in each hospital. OMOP CDM generates network-wide results through distributed research networks using the same analysis program among collaborating organizations^42^.

The EHRs from (1) the AUMC (2,873,443 patients; dated between January 1994 and February 2022), (2) Gyeongsang National University Hospital (626,663 patients; dated between October 2009 and April 2022), (3) Kangdong Sacred Heart Hospital (1,724,052 patients; dated between October 1986 and December 2019), (4) Kyunghee University Medical Center (1,168,640 patients; dated between January 2008 and February 2022), (5) Pusan National University Hospital (791,935 patients; dated between February 2011 and August 2019), (6) Soonchunhyang University Bucheon Hospital (1,301,117 patients; dated between February 2001 and May 2021), and (7) Soonchunhyang University Cheonan Hospital (987,701 patients; dated between February 2006 and May 2021) were converted to CDM data. The converted EHRs included the following information: diagnostic codes generated from all types of examination reports, including outpatient, inpatient, and medication data, and conversions of text-based reports^43^.

### Study design

We conducted a multicenter, retrospective, observational, comparative cohort study using a distributed research network without patient-level data sharing. We extracted 4,669 adult patients with HNC aged > 18 years who had records that underwent surgery for more than 180 days postoperatively. The study population included oral cavity cancers, oropharyngeal, hypopharyngeal, nasopharyngeal cancers, and laryngeal cancers. We included patients with esophageal cancers in the HNC group. Cancers of salivary glands were excluded because of the different pathologic types.

We divided patients into two groups: (1) statin users, who took statins continuously for at least 180 days within 5 years postoperatively (target cohorts), and (2) non-users, who did not fulfill the aforementioned criterion (control cohorts). Statin use included uninterrupted use of rosuvastatin, atorvastatin, pitavastatin, simvastatin, pravastatin, fluvastatin, and lovastatin for more than 6 months throughout the observation period for each patient. This includes both the period before a patient is diagnosed with HNC and within 5 years of diagnosis.

We defined the index date as the date of surgery occurring between 90 days prior to diagnosis and 14 days following diagnosis and excluded patients who did not have observational records spanning at least 180 days. The primary outcome was HNC recurrence at 3 and 5 years after the index date. Recurrence was defined as surgery, chemotherapy, and radiotherapy within 6 months postoperatively to treat HNC. The secondary outcome was overall death 3 and 5 years after the index date.

To demonstrate the robustness of the study, we performed four subgroup analyses: (1) one in which cases of esophageal cancer were excluded, (2) one of individuals aged over 65 years (elderly), (3) one of males, and (4) one according to statin type (atorvastatin and rosuvastatin).

### Statistical analysis

Data were analyzed using ATLAS version 2.7.6, an interactive analysis platform, and FEEDER-NET, a health big data platform based on OMOP-CDM. We performed 1:4 propensity score-adjusted matching using covariates such as age, gender, and Romano’s Adaptation of the Charlson Comorbidity Index (CCI)^44,45^, a widely used method for categorizing comorbidities to predict various cancers' short- and long-term mortality from medical records^46–49^. Romano’s Adaptation of the CCI demonstrated better performance in predicting short- and long-term mortality than standard CCI and was previously used to assess comorbidities in patients with HNC^49^. The width of the caliper was 0.2 standardized logits. Age covariates were grouped by an age of five years. After propensity score-adjusted matching, we conducted a Cox regression analysis to examine the hazard ratio with 95% CIs. We tested the proportional hazard assumption with the scaled Schoenfeld residuals test. The Scaled Schoenfeld residuals test is a proportional hazard assumption test commonly used in Cox regression^50,51^. A *p*-value over 0.05 indicates that the hazard ratio is stable between groups and does not vary significantly over time. We utilized the Kaplan–Meier plot to visually compare the probability of recurrence between statin users and non-users over a 5-year period following the index date. To assess the statistical significance of any differences between both groups, we performed a log-rank test and calculated the *p*-value, indicating the likelihood of observing the differences between the groups randomly^52^. After performing the same analysis process using the R package for each of the 7 databases (The R Foundation for Statistical Computing, Vienna, Austria), we meta-analyzed their aggregated results. Statistical tests for heterogeneity across studies were assessed using *x*^2^ and *I*^2^ statistics. We used fixed- and random-effect models^42^, reporting fixed-effect model meta-analysis results when *I*^2^ < 50% and random-effect model meta-analysis results when *I*^2^ > 50%. All analyses were performed using R (The R Foundation, Vienna, Austria) and the following R packages: CohortMethod^53^, SelfControlledCaseSeries^54^, SelfControlledCohort^55^, and EvidenceSynthesis^56^ version 4.1.0 (The R foundation).

### Ethical statement

This study was approved by the Institutional Review Board (IRB) of the Ajou University Medical Center (approval number: AJOUIRB-MDB-2021–700) and was allowed to waive the requirement to obtain informed consent. The other six hospitals are affiliated with the Research Border Free Zoon of Korea, which accepts IRB approval of the research organizing center for studies using unidentified CDM data. This study was performed in accordance with the principles of the Helsinki Declaration, as revised in 2013.

### Consent to participate

The Requirement of informed consent was waived because of the retrospective nature of this study.

### Supplementary Information

Supplementary Figures.

Supplementary Tables.

## Data Availability

The datasets generated in this study are included in this published article and its supplementary files.
