# Supplementary material for: Effect of statin use on head and neck cancer prognosis in a multicenter study using a Common Data Model
Source: Sci Rep. 2023 Nov 13;13:19770. doi: 10.1038/s41598-023-45654-7 (PMC10643676; doi:10.1038/s41598-023-45654-7)
Supplement: Supplementary file 1 — Supplementary Figures. [file 41598_2023_45654_MOESM1_ESM.pdf]

Supplementary Figure 1

(A) 3-year mortality

Hospital	Statin-users: Death	Statin-users: Non-death	Non-users: Death	Non-users: Non-death	Risk Ratio [95% CI]
AUMC	4	64	26	225	0.57 [0.21, 1.57]
GNUH	7	38	23	157	1.22 [0.56, 2.66]
KDH	4	48	23	185	0.70 [0.25, 1.92]
KHMC	2	41	19	153	0.42 [0.10, 1.74]
PNUH	2	47	11	185	0.73 [0.17, 3.17]
SCHBC	1	36	14	134	0.29 [0.04, 2.10]
SCHCA	0	42	10	158	0.19 [0.01, 3.13]
Fixed Model (Q = 4.20, df = 6, p = 0.65; I^2 = 0.0%)					0.71 [0.45, 1.12]

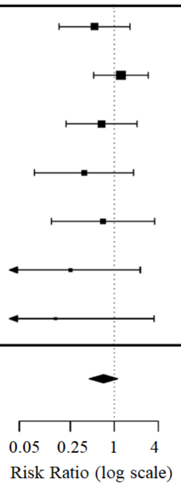

(B) 5-year mortality

Hospital	Statin-users: Death	Statin-users: Non-death	Non-users: Death	Non-users: Non-death	Risk Ratio [95% CI]
AUMC	6	62	28	223	0.79 [0.34, 1.83]
GNUH	8	37	25	155	1.28 [0.62, 2.65]
KDH	7	45	26	182	1.08 [0.50, 2.34]
KHMC	2	41	19	153	0.42 [0.10, 1.74]
PNUH	2	47	12	184	0.67 [0.15, 2.88]
SCHBC	1	36	18	130	0.22 [0.03, 1.61]
SCHCA	1	41	14	154	0.29 [0.04, 2.11]
Fixed Model (Q = 5.56, df = 6, p = 0.47; I^2 = 0.0%)					0.85 [0.57, 1.26]

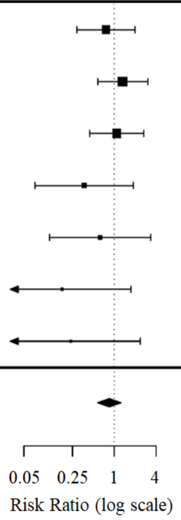

Supplementary Figure 1. Meta-analysis of mortality and statin use.

Supplementary Figure 2-1

(A)

Hospital	Statin-users: Recurrence	Statin-users: Non-recurrence	Non-users: Recurrence	Non-users: Non-recurrence	Risk Ratio [95% CI]
AUMC	5	44	18	178	1.11 [0.43, 2.84]
GNUH	7	35	22	146	1.27 [0.58, 2.78]
KDH	6	44	30	170	0.80 [0.35, 1.82]
KHMC	2	35	19	129	0.42 [0.10, 1.73]
PNUH	1	41	12	156	0.33 [0.04, 2.49]
SCHBC	1	30	17	107	0.24 [0.03, 1.70]
SCHCA	1	34	13	127	0.31 [0.04, 2.27]
Fixed Model (Q = 5.70, df = 6, p = 0.46; I^2 = 0.0%)					0.81 [0.53, 1.23]

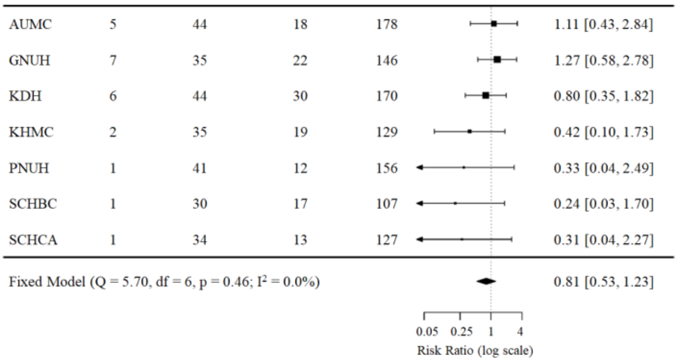

(B)

Hospital	Statin-users: Recurrence	Statin-users: Non-recurrence	Non-users: Recurrence	Non-users: Non-recurrence	Risk Ratio [95% CI]
AUMC	10	44	45	171	0.89 [0.48, 1.65]
GNUH	6	31	25	123	0.96 [0.42, 2.17]
KDH	11	39	35	165	1.26 [0.69, 2.30]
KHMC	4	28	33	95	0.48 [0.19, 1.27]
PNUH	6	33	27	129	0.89 [0.39, 2.00]
SCHBC	6	22	23	82	0.98 [0.44, 2.17]
SCHCA	8	30	44	108	0.73 [0.37, 1.41]
Fixed Model (Q = 3.23, df = 6, p = 0.78; I^2 = 0.0%)					0.90 [0.68, 1.18]

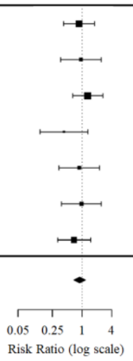

(C)

Hospital	Statin-users: Recurrence	Statin-users: Non-recurrence	Non-users: Recurrence	Non-users: Non-recurrence	Risk Ratio [95% CI]
AUMC	7	29	24	120	1.17 [0.55, 2.49]
GNUH	3	18	12	72	1.00 [0.31, 3.23]
KDH	6	12	7	65	3.43 [1.31, 8.96]
KHMC	1	18	17	59	0.24 [0.03, 1.66]
PNUH	2	24	19	85	0.42 [0.10, 1.69]
SCHBC	2	8	2	38	4.00 [0.64, 25.02]
SCHCA	2	15	16	52	0.50 [0.13, 1.97]
Random Model (Q = 12.75, df = 6, p = 0.05; I^2 = 53.4%)					1.06 [0.53, 2.11]

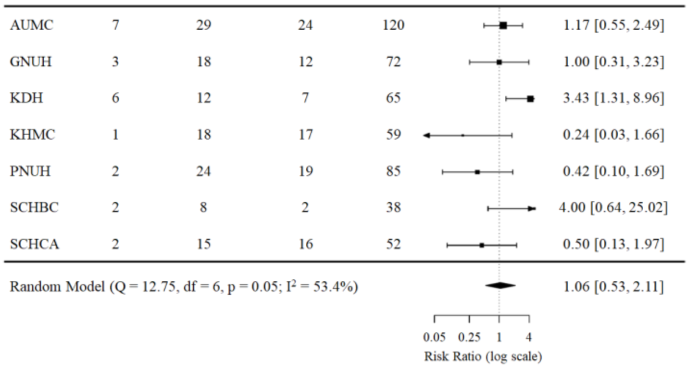

(D)

Hospital	Statin-users: Recurrence	Statin-users: Non-recurrence	Non-users: Recurrence	Non-users: Non-recurrence	Risk Ratio [95% CI]
AUMC	8	46	41	175	0.78 [0.39, 1.57]
GNUH	5	32	23	125	0.87 [0.35, 2.13]
KDH	10	40	33	167	1.21 [0.64, 2.29]
KHMC	2	30	29	99	0.28 [0.07, 1.10]
PNUH	2	37	27	129	0.30 [0.07, 1.19]
SCHBC	6	22	20	85	1.12 [0.50, 2.53]
SCHCA	6	32	43	109	0.56 [0.26, 1.21]
Fixed Model (Q = 7.42, df = 6, p = 0.28; I^2 = 19.1%)					0.79 [0.58, 1.09]

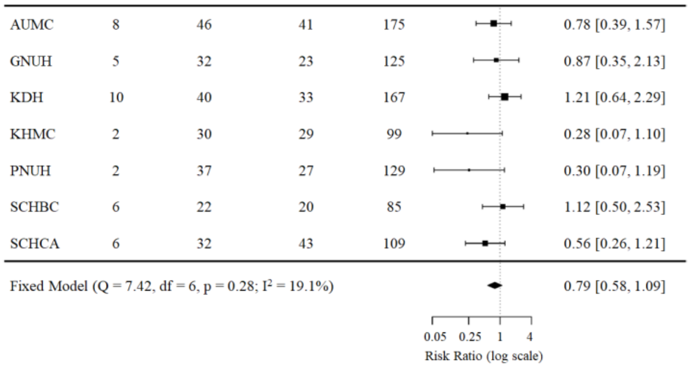

(E)

Hospital	Statin-users: Recurrence	Statin-users: Non-recurrence	Non-users: Recurrence	Non-users: Non-recurrence	Risk Ratio [95% CI]
AUMC	12	37	45	151	1.07 [0.61, 1.86]
GNUH	7	35	24	144	1.17 [0.54, 2.52]
KDH	11	39	36	164	1.22 [0.67, 2.23]
KHMC	3	34	37	111	0.32 [0.11, 0.99]
PNUH	2	40	41	127	0.20 [0.05, 0.77]
SCHBC	8	23	33	91	0.97 [0.50, 1.88]
SCHCA	6	29	44	96	0.55 [0.25, 1.18]
Fixed Model (Q = 11.33, df = 6, p = 0.08; I^2 = 47.0%)					0.87 [0.66, 1.15]

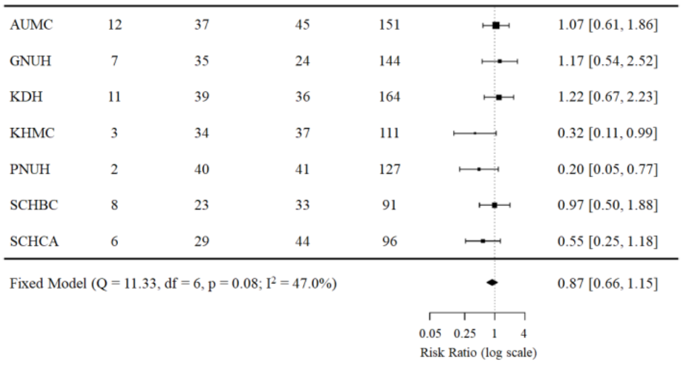

(F)

Hospital	Statin-users: Recurrence	Statin-users: Non-recurrence	Non-users: Recurrence	Non-users: Non-recurrence	Risk Ratio [95% CI]
AUMC	13	36	49	147	1.06 [0.63, 1.79]
GNUH	8	34	26	142	1.23 [0.60, 2.52]
KDH	11	39	37	163	1.19 [0.65, 2.16]
KHMC	5	32	44	104	0.45 [0.19, 1.07]
PNUH	6	36	45	123	0.53 [0.24, 1.17]
SCHBC	9	22	36	88	1.00 [0.54, 1.85]
SCHCA	7	28	45	95	0.62 [0.31, 1.26]
Fixed Model (Q = 7.28, df = 6, p = 0.30; I^2 = 17.6%)					0.88 [0.69, 1.14]

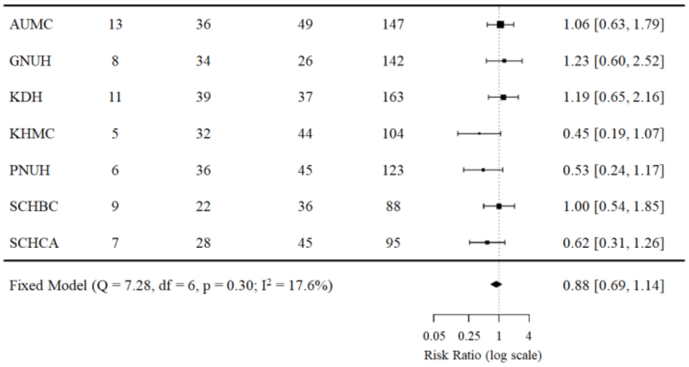

Supplementary Figure 2. Meta-analysis of the comparison of recurrence rates between statin users and non-users in subgroups. (A) Excluding esophageal cancer 3-year recurrence. (B) Excluding esophageal cancer 5-year recurrence. (C) Elder 3-year recurrence. (D) Elder 5-year recurrence. (E) Male 3-year recurrence. (F) Male 5-year recurrence. (G) Atorvastatin 3-year recurrence. (H) Atorvastatin 5-year recurrence. (I) Rosuvastatin 3-year recurrence. (J) Rosuvastatin 5-year recurrence.

Supplementary Figure 2-2

(G)

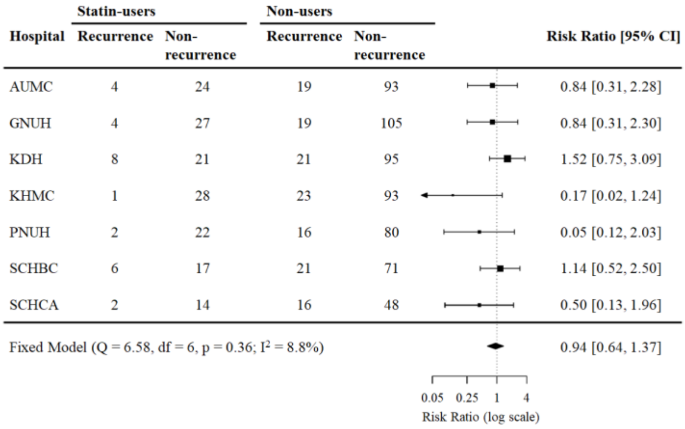

Hospital	Statin-users Recurrence	Statin-users Non-recurrence	Non-users Recurrence	Non-users Non-recurrence	Risk Ratio [95% CI]
AUMC	4	24	19	93	0.84 [0.31, 2.28]
GNUH	4	27	19	105	0.84 [0.31, 2.30]
KDH	8	21	21	95	1.52 [0.75, 3.09]
KHMC	1	28	23	93	0.17 [0.02, 1.24]
PNUH	2	22	16	80	0.05 [0.12, 2.03]
SCHBC	6	17	21	71	1.14 [0.52, 2.50]
SCHCA	2	14	16	48	0.50 [0.13, 1.96]
Fixed Model (Q = 6.58, df = 6, p = 0.36; I² = 8.8%)					0.94 [0.64, 1.37]

(H)

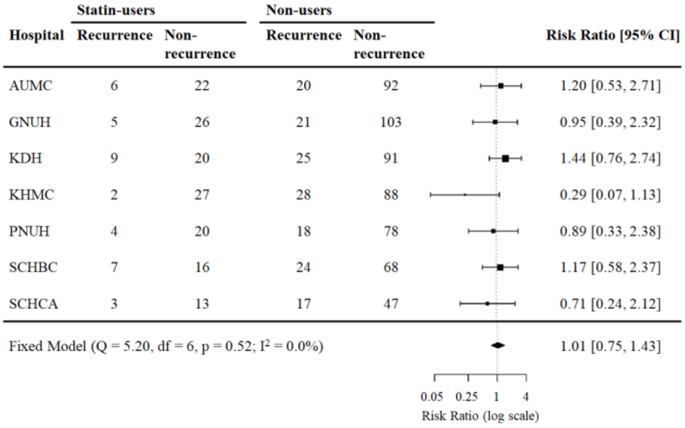

Hospital	Statin-users Recurrence	Statin-users Non-recurrence	Non-users Recurrence	Non-users Non-recurrence	Risk Ratio [95% CI]
AUMC	6	22	20	92	1.20 [0.53, 2.71]
GNUH	5	26	21	103	0.95 [0.39, 2.32]
KDH	9	20	25	91	1.44 [0.76, 2.74]
KHMC	2	27	28	88	0.29 [0.07, 1.13]
PNUH	4	20	18	78	0.89 [0.33, 2.38]
SCHBC	7	16	24	68	1.17 [0.58, 2.37]
SCHCA	3	13	17	47	0.71 [0.24, 2.12]
Fixed Model (Q = 5.20, df = 6, p = 0.52; I² = 0.0%)					1.01 [0.75, 1.43]

(I)

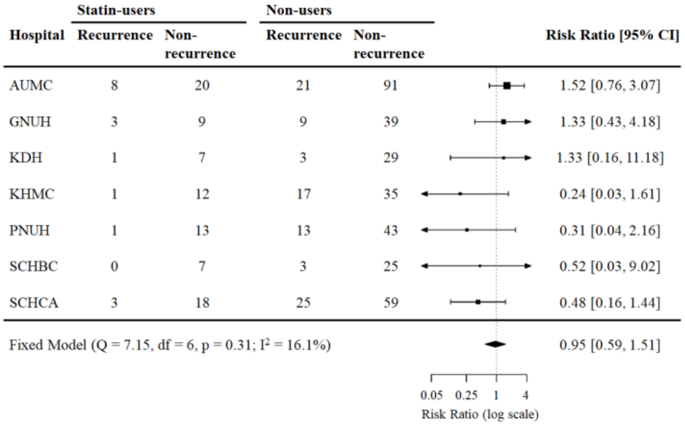

Hospital	Statin-users Recurrence	Statin-users Non-recurrence	Non-users Recurrence	Non-users Non-recurrence	Risk Ratio [95% CI]
AUMC	8	20	21	91	1.52 [0.76, 3.07]
GNUH	3	9	9	39	1.33 [0.43, 4.18]
KDH	1	7	3	29	1.33 [0.16, 11.18]
KHMC	1	12	17	35	0.24 [0.03, 1.61]
PNUH	1	13	13	43	0.31 [0.04, 2.16]
SCHBC	0	7	3	25	0.52 [0.03, 9.02]
SCHCA	3	18	25	59	0.48 [0.16, 1.44]
Fixed Model (Q = 7.15, df = 6, p = 0.31; I² = 16.1%)					0.95 [0.59, 1.51]

(J)

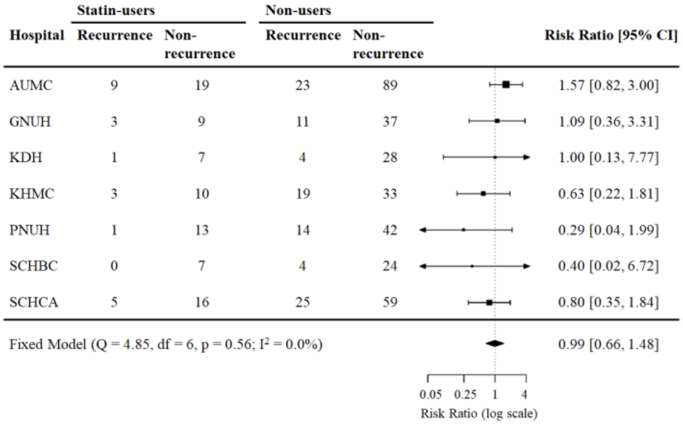

Hospital	Statin-users Recurrence	Statin-users Non-recurrence	Non-users Recurrence	Non-users Non-recurrence	Risk Ratio [95% CI]
AUMC	9	19	23	89	1.57 [0.82, 3.00]
GNUH	3	9	11	37	1.09 [0.36, 3.31]
KDH	1	7	4	28	1.00 [0.13, 7.77]
KHMC	3	10	19	33	0.63 [0.22, 1.81]
PNUH	1	13	14	42	0.29 [0.04, 1.99]
SCHBC	0	7	4	24	0.40 [0.02, 6.72]
SCHCA	5	16	25	59	0.80 [0.35, 1.84]
Fixed Model (Q = 4.85, df = 6, p = 0.56; I² = 0.0%)					0.99 [0.66, 1.48]

Supplementary Figure 2. Meta-analysis of the comparison of recurrence rates between statin users and non-users in subgroups. (A) Excluding esophageal cancer 3-year recurrence. (B) Excluding esophageal cancer 5-year recurrence. (C) Elder 3-year recurrence. (D) Elder 5-year recurrence. (E) Male 3-year recurrence. (F) Male 5-year recurrence. (G) Atorvastatin 3-year recurrence. (H) Atorvastatin 5-year recurrence. (I) Rosuvastatin 3-year recurrence. (J) Rosuvastatin 5-year recurrence.

Supplementary Figure 3

(A) 3-year Recurrence

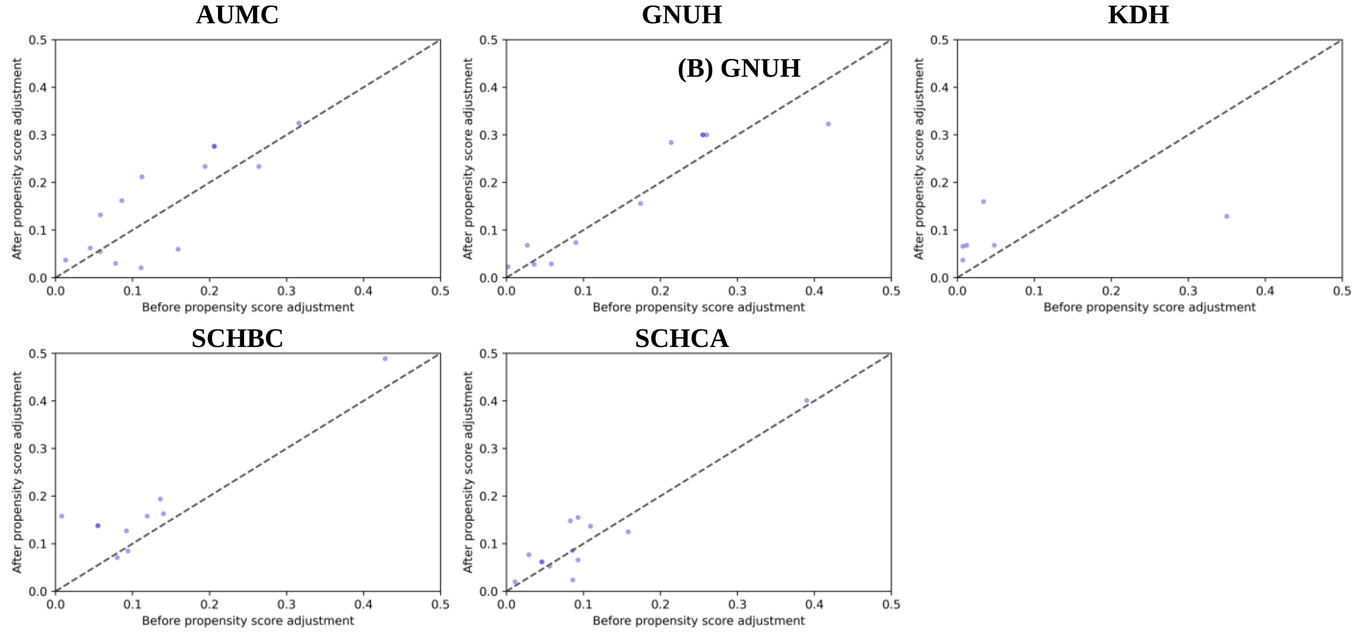

(B) 5-year Recurrence

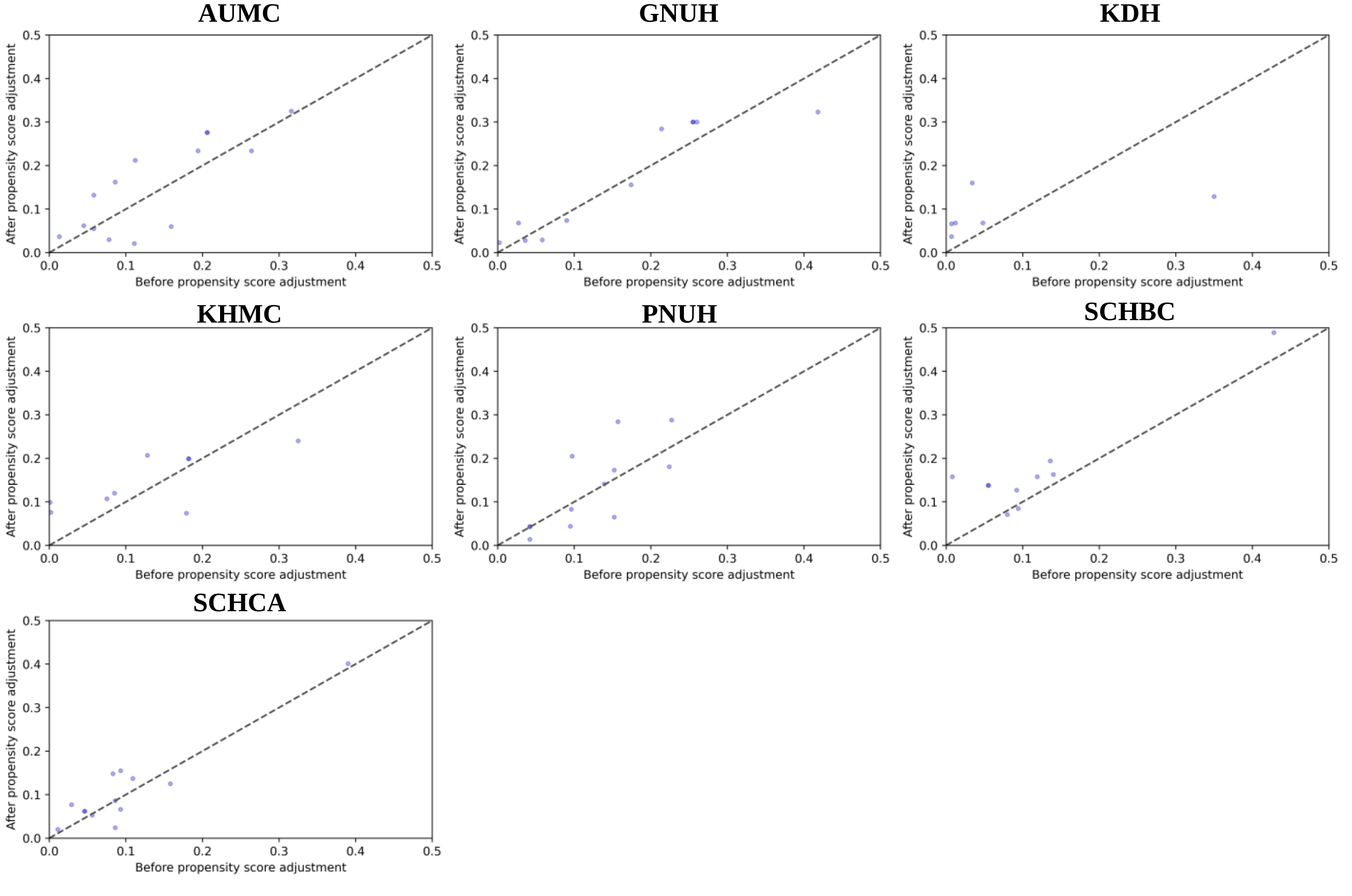

Supplementary 3 Covariate balance before and after propensity score adjustment.
