# Supplementary material for: Effect of statin use on head and neck cancer prognosis in a multicenter study using a Common Data Model
Source: Sci Rep. 2023 Nov 13;13:19770. doi: 10.1038/s41598-023-45654-7 (PMC10643676; doi:10.1038/s41598-023-45654-7)
Supplement: Supplementary file 2 — Supplementary Tables. [file 41598_2023_45654_MOESM2_ESM.docx]

**Supplementary Table 1.** Demographic and clinical characteristics of the unmatched and propensity score-matched AUMC cohort.

|  | **Before PS adjustment** | | | **After PS adjustment** | | |
| --- | --- | --- | --- | --- | --- | --- |
|  | **Stain-users  (n = 68)** | **Non-users (n = 1151)** | **p-value** | **Stain-users (n = 68)** | **Non-users (n = 251)** | **p-value** |
| **Age group** |  |  | 0.020 |  |  | 1.000 |
| 18-19 | 0 ( 0.0%) | 2 ( 0.2%) |  | 0 ( 0.0%) | 0 ( 0.0%) |  |
| 20-29 | 0 ( 0.0%) | 19 ( 1.7%) |  | 0 ( 0.0%) | 3 ( 1.2%) |  |
| 30-39 | 2 ( 2.9%) | 46 ( 4.0%) |  | 2 ( 2.9%) | 9 ( 3.6%) |  |
| 40-49 | 3 ( 4.4%) | 150 (13.0%) |  | 3 ( 4.4%) | 35 (13.9%) |  |
| 50-59 | 16 (23.5%) | 283 (24.6%) |  | 16 (23.5%) | 60 (23.9%) |  |
| 60-69 | 17 (25.0%) | 349 (30.3%) |  | 17 (25.0%) | 81 (32.3%) |  |
| 70-79 | 20 (29.4%) | 231 (20.1%) |  | 20 (29.4%) | 49 (19.5%) |  |
| 80-89 | 10 (14.7%) | 63 ( 5.5%) |  | 10 (14.7%) | 12 ( 4.8%) |  |
| 90-99 | 0 ( 0.0%) | 8 ( 0.7%) |  | 0 ( 0.0%) | 2 ( 0.8%) |  |
| **Gender** |  |  | 0.113 |  |  | 0.068 |
| Female | 19 (27.9%) | 222 (19.3%) |  | 19 (27.9%) | 43 (17.1%) |  |
| male | 49 (72.1%) | 929 (80.7%) |  | 49 (72.1%) | 208 (82.9%) |  |
| **Charlson comorbidity index** |  |  |  |  |  |  |
| Hypertension | 41 (60.3%) | 261 (22.7%) | <0.001 | 41 (60.3%) | 66 (26.3%) | <0.001 |
| acute myocardial infarction | 4 ( 5.9%) | 1 ( 0.1%) | <0.001 | 4 ( 5.9%) | 0 ( 0.0%) | 0.001 |
| Congestive heart failure | 3 ( 4.4%) | 10 ( 0.9%) | 0.031 | 3 ( 4.4%) | 6 ( 2.4%) | 0.631 |
| Peripheral vascular disease | 1 ( 1.5%) | 3 ( 0.3%) | 0.546 | 1 ( 1.5%) | 1 ( 0.4%) | 0.898 |
| Cerebrovascular disease | 6 ( 8.8%) | 11 ( 1.0%) | <0.001 | 6 ( 8.8%) | 3 ( 1.2%) | 0.003 |
| Dementia | 2 ( 2.9%) | 4 ( 0.3%) | 0.038 | 2 ( 2.9%) | 2 ( 0.8%) | 0.426 |
| Chronic pulmonary disease | 6 ( 8.8%) | 85 ( 7.4%) | 0.841 | 6 ( 8.8%) | 27 (10.8%) | 0.810 |
| Rheumatologic disease | 1 ( 1.5%) | 3 ( 0.3%) | 0.546 | 1 ( 1.5%) | 2 ( 0.8%) | 1.000 |
| Peptic ulcer disease | 3 ( 4.4%) | 31 ( 2.7%) | 0.647 | 3 ( 4.4%) | 7 ( 2.8%) | 0.773 |
| Mild liver disease | 1 ( 1.5%) | 21 ( 1.8%) | 1.000 | 1 ( 1.5%) | 10 ( 4.0%) | 0.527 |
| Diabetes | 13 (19.1%) | 94 ( 8.2%) | 0.004 | 13 (19.1%) | 29 (11.6%) | 0.152 |
| Diabetes with chronic complications | 5 ( 7.4%) | 15 ( 1.3%) | 0.001 | 5 ( 7.4%) | 6 ( 2.4%) | 0.106 |
| Hemoplegia or paralegia | 0 ( 0.0%) | 1 ( 0.1%) | 1.000 | 0 ( 0.0%) | 0 ( 0.0%) | 1.000 |
| Renal disease | 4 ( 5.9%) | 24 ( 2.1%) | 0.106 | 4 ( 5.9%) | 10 ( 4.0%) | 0.731 |
| Any malignancy | 67 (98.5%) | 1145 (99.5%) | 0.856 | 67 (98.5%) | 251 (100.0%) | 0.483 |
| Moderate to severe liver disease | 0 ( 0.0%) | 5 ( 0.4%) | 1.000 | 0 ( 0.0%) | 4 ( 1.6%) | 0.665 |
| Metastatic solid tumor | 23 (33.8%) | 458 (39.8%) | 0.395 | 23 (33.8%) | 100 (39.8%) | 0.445 |
| AIDS | 0 ( 0.0%) | 0 ( 0.0%) |  | 0 ( 0.0%) | 0 ( 0.0%) |  |

PS, propensity score, SMD, Standardized Mean Difference

**Supplementary Table 2.** Results of scaled Schoenfeld residuals test for testing proportional hazard assumption. Each alphabet means Ajou University Medical Center, AUMC, Gyeongsang National University Hospital, GNUH, Kangdong Sacred Heart Hospital, KDH, Kyunghee University Medical Center, KHMC, Pusan National University Hospital, PNUH, Soonchunhyang University Bucheon Hospital, SCHBC and Soonchunhyang University Cheonan Hospital, SCHCA.

| **Hospital** | **scaled Schoenfeld residuals** | **p-value** |
| --- | --- | --- |
| AUMC | -0.188 | 0.021 |
| GNUH | -0.177 | 0.743 |
| KDH | -0.170 | 0.472 |
| KHMC | -0.087 | 0.636 |
| PNUH | 0.873 | 0.365 |
| SCHBC | -0.167 | 0.799 |
| SCHCA | -0.119 | 0.642 |
